# Supplementary material for: Assessing sequence-based protein–protein interaction predictors for use in therapeutic peptide engineering
Source: Sci Rep. 2022 Jun 10;12:9610. doi: 10.1038/s41598-022-13227-9 (PMC9187631; doi:10.1038/s41598-022-13227-9)
Supplement: Supplementary file 1 — Supplementary Information. [file 41598_2022_13227_MOESM1_ESM.pdf]

Assessing sequence-based protein-protein interaction predictors for use in therapeutic peptide engineering

François Charih, Kyle K. Biggar, James R. Green

Supplementary figures

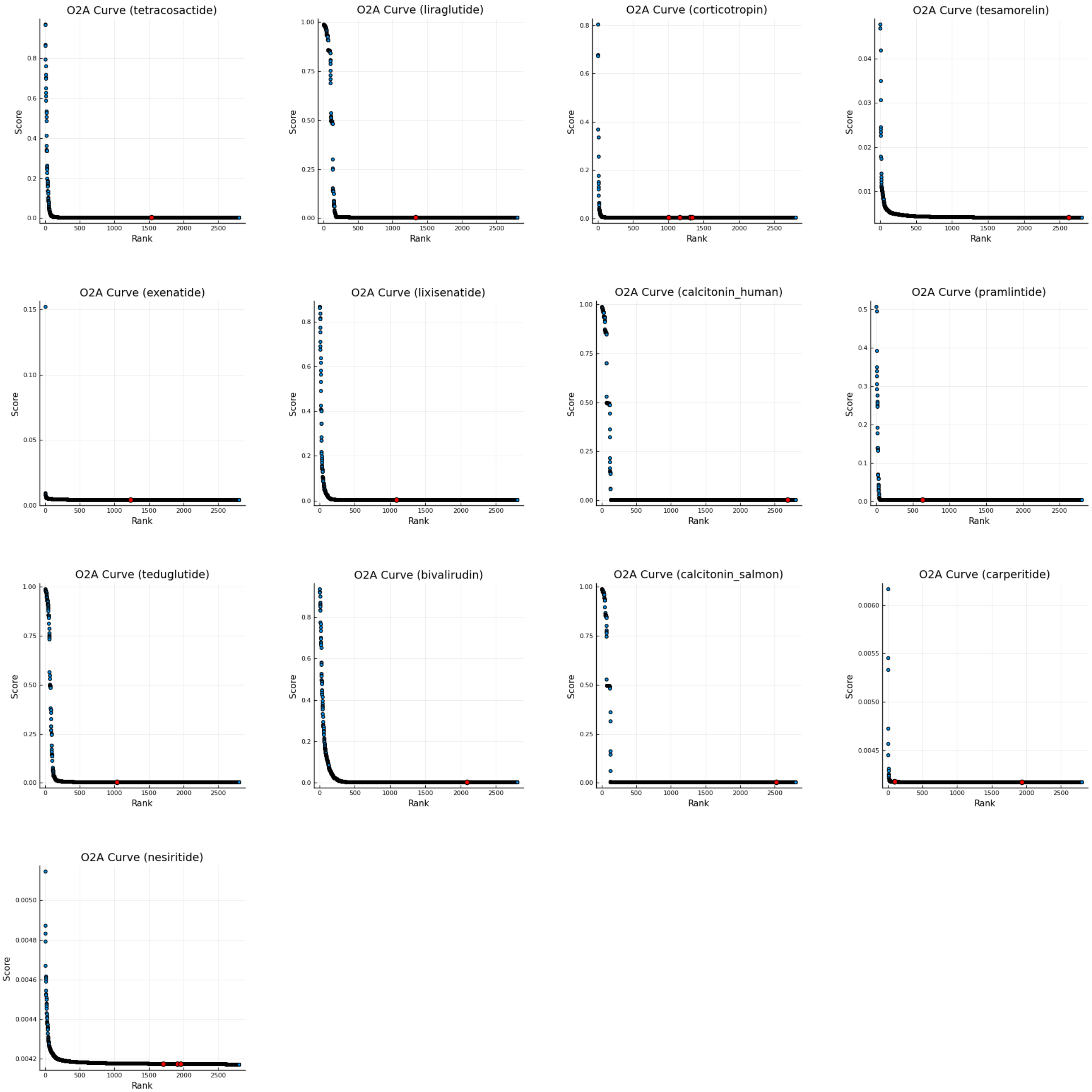

Supplementary Figure 1. **All one-to-all curves generated with D-SCRIPT.**

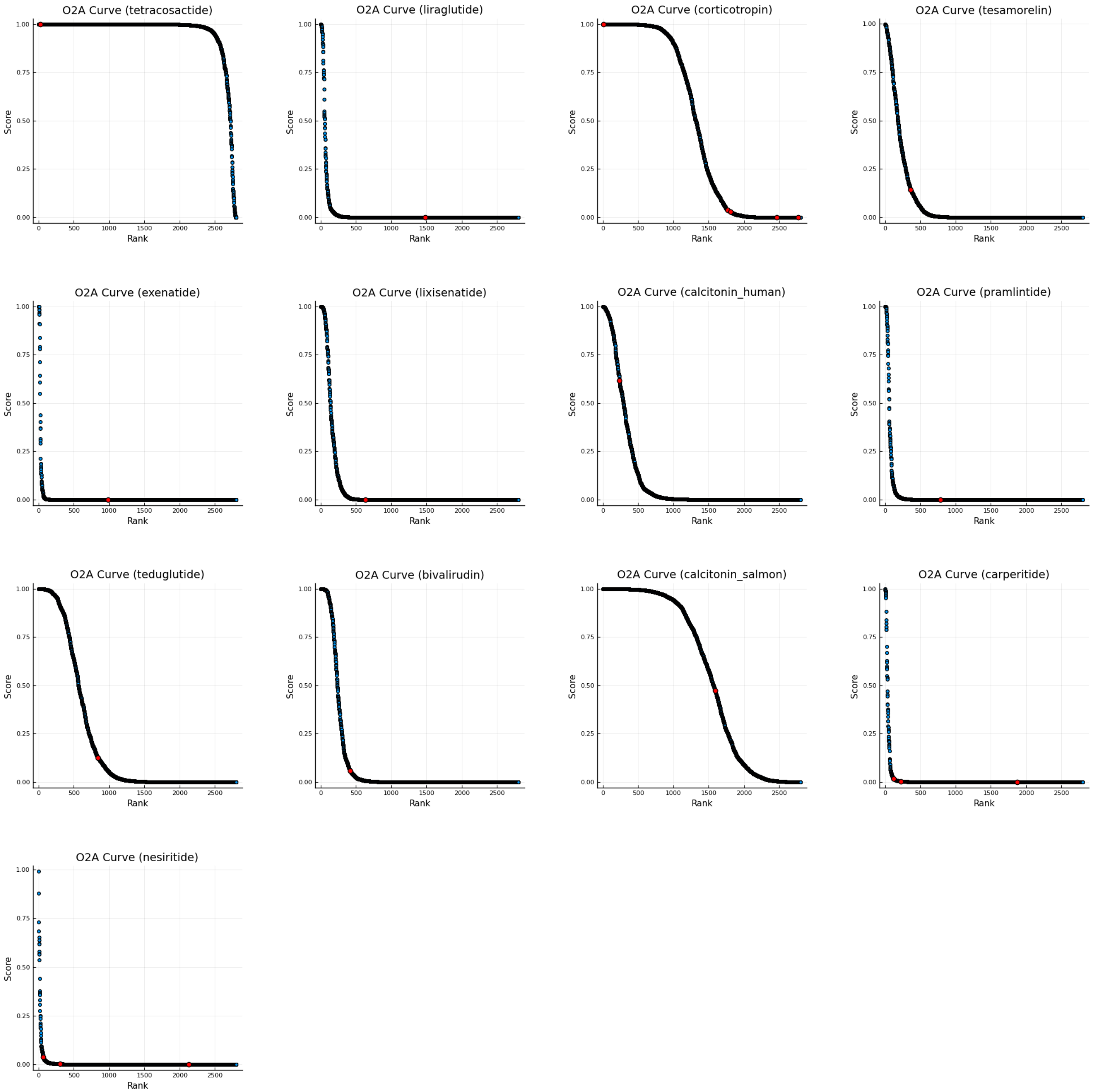

Supplementary Figure 2. **All one-to-all curves generated with PIPR (optimistic scenario).**

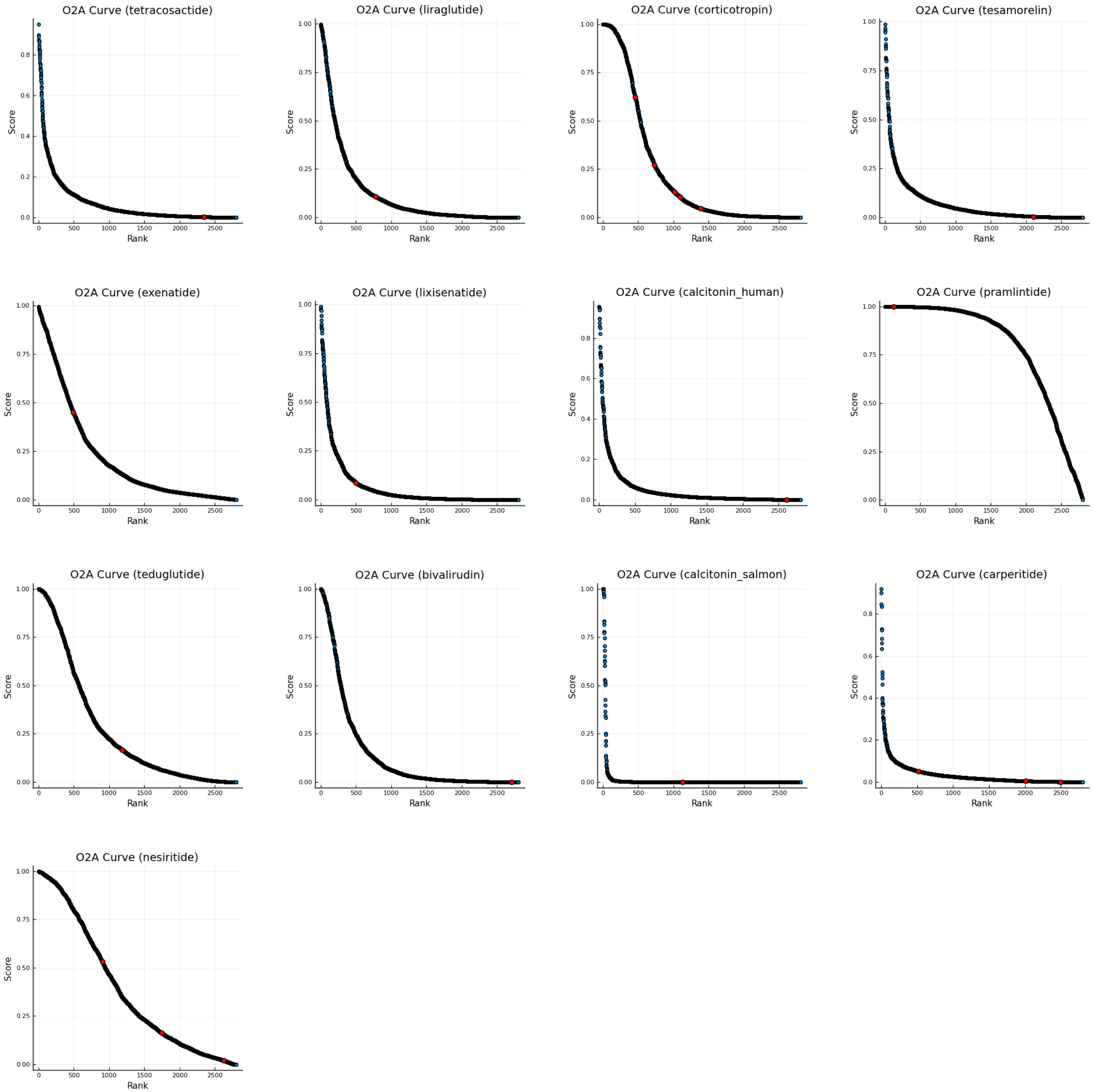

Supplementary Figure 3. **All one-to-all curves generated with PIPR (pessimistic scenario).**

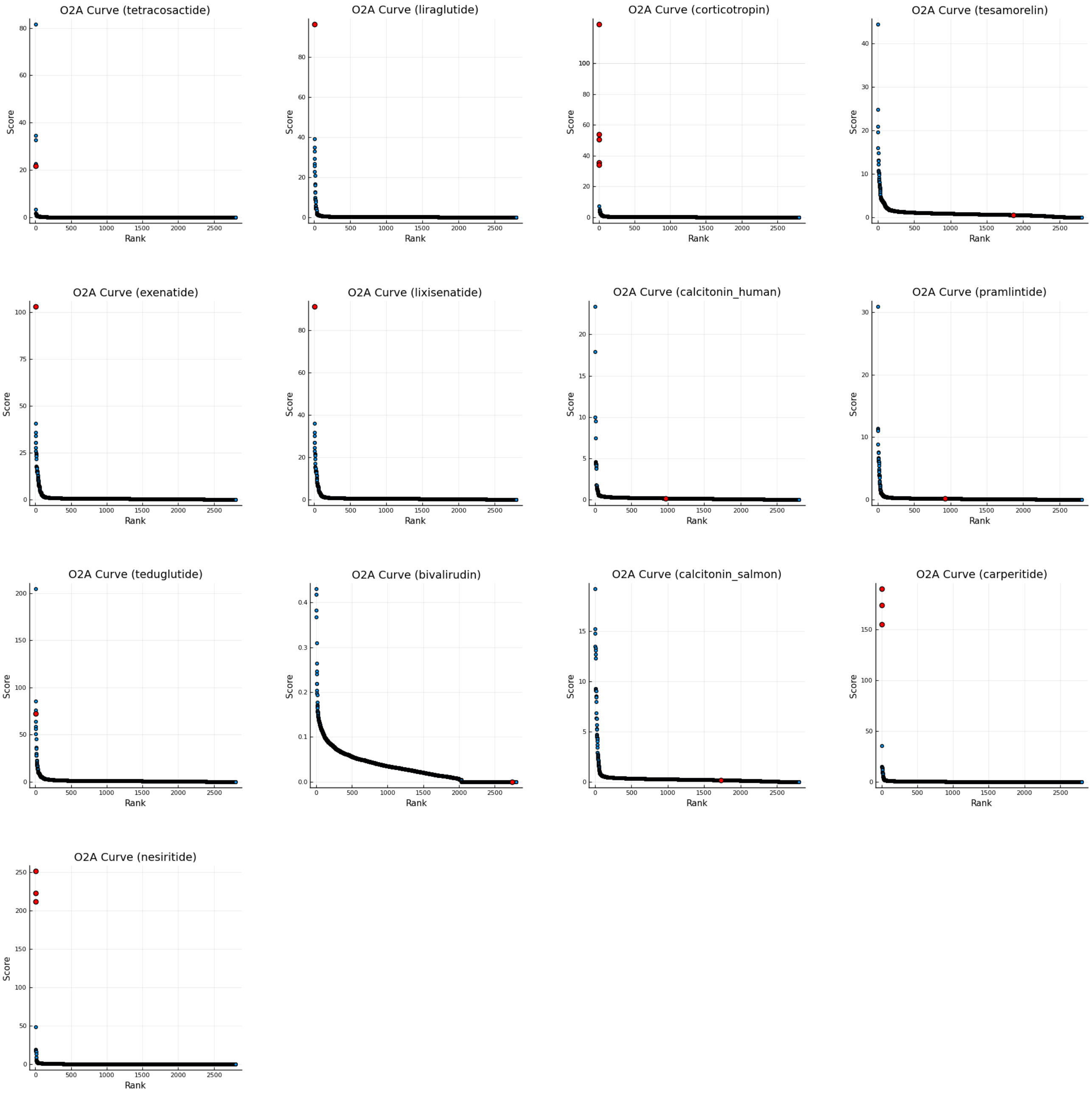

Supplementary Figure 4. All one-to-all curves generated with SPRINT (optimistic scenario).

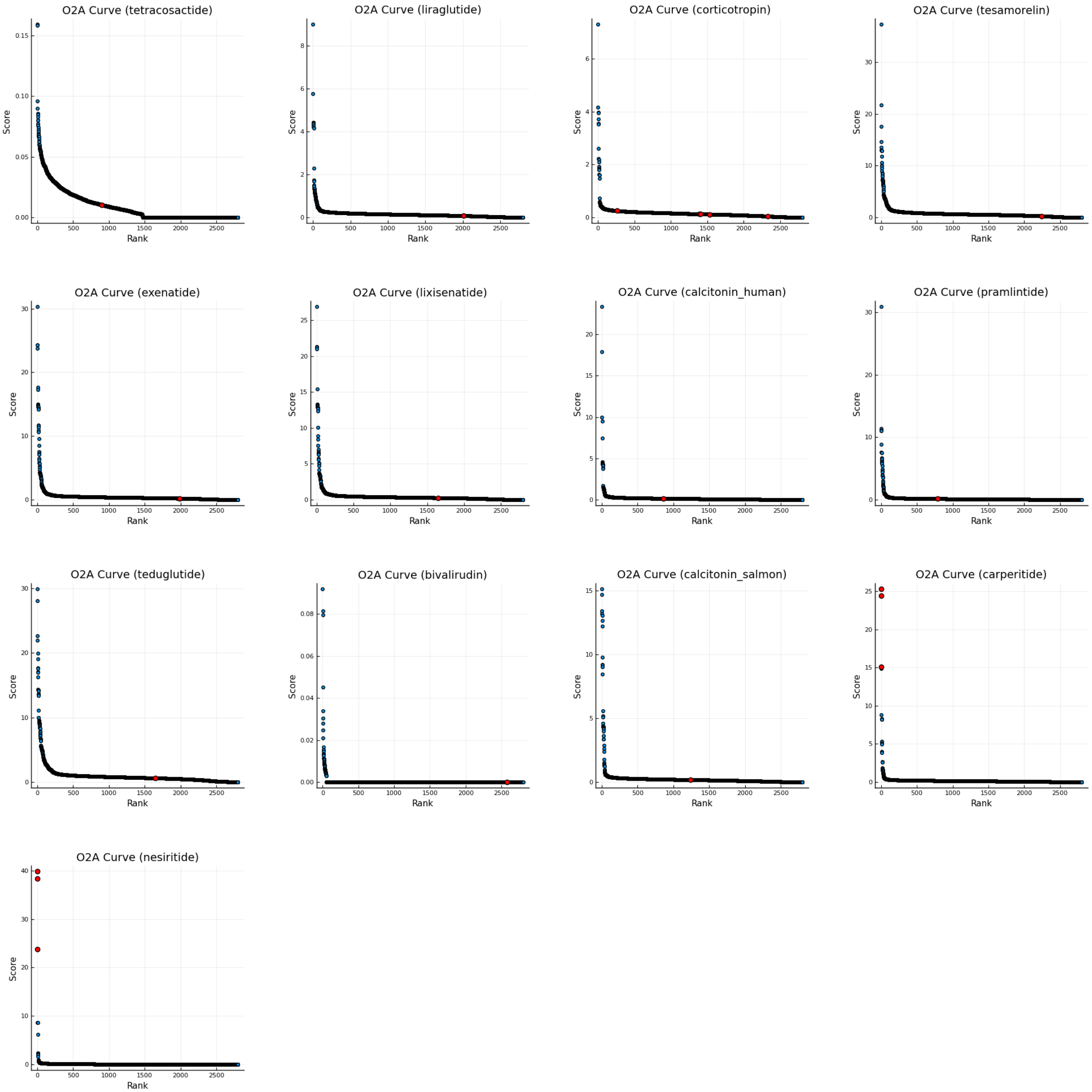

Supplementary Figure 5. **All one-to-all curves generated with SPRINT (pessimistic scenario).**
